# Impact of Nutrients on Protozoa Community Diversity and Structure in Litter of Two Natural Grass Species in a Copper Tailings Dam, China

**DOI:** 10.3390/microorganisms9112250

**Published:** 2021-10-28

**Authors:** Tong Jia, Xiaoxia Liang, Tingyan Guo, Baofeng Chai

**Affiliations:** Institute of Loess Plateau, Shanxi University, Taiyuan 030006, China; liangxiaoxia2020@163.com (X.L.); paper_submit_2020@163.com (T.G.); bfchai@sxu.edu.cn (B.C.)

**Keywords:** copper tailings dam, litter, protozoan community, diversity, *Bothriochloa ischaemum*, *Imperata cylindrica*

## Abstract

In nature, protists directly participate in litter decomposition and indirectly affect litter decomposition processes by means of their influence on litter microbial communities. To date, relevant studies on litter microbial communities have primarily focused on bacteria and fungi, while relatively little attention has been paid to the characteristics of protozoan communities within damaged ecosystems. Two dominant grass species (*Bothriochloa ischaemum* and *Imperata cylindrica*) were selected from China’s “Eighteenth” River tailings dam to explore protozoan community composition and diversity in a degraded mining area and to clarify the influence among key ecological factors and protozoan community characteristics in litter. High-throughput sequencing was used to analyze protozoan community composition and diversity, while correlation analysis was used to explore the relationships between protozoan communities and litter nutrient characteristics, including associative enzyme degradation. Although protozoan communities in litter shared a dominant group at an order level (Colpodida), they differed at a genus level (i.e., *Hausmanniella* and *Tychosporium*). Moreover, although the order Cryomonadida positively correlated to total nitrogen (TN) and sucrose, it exhibited an extreme negative correlation to total carbon (TC) and cellulase. Colpodida and Oomycota_X significantly and negatively correlated to litter urease activity. Nutrient characteristics of grass litter in copper tailing dams are important ecological factors that affect protozoan community characteristics. Notable differences were observed among protozoan communities of these two grass species, while litter enzyme activities were closely correlated to protozoan community diversity. The results suggested that Colpodida may play important roles in litter decomposition and nutrient cycling in mining areas.

## 1. Introduction

Litter acts as a pathway for plants to transfer nutrients to the soil within terrestrial ecosystems [1]. Greater than 90% of the net production of aboveground plant components are returned to soil via litter [2]. Moreover, the carbon (C) released through litter decomposition is an important source of soil organic matter conversion into humus, which is highly significant for material circulation. Protists are an integral constituent of soil microbial communities [2]. Autotrophic protists provide vital C inputs to soil [3], and certain protists also participate in organic matter degradation processes [4]. In addition, protists are major consumers within soil food webs, which regulate microbial communities through phagocytosis or the secretion of metabolites [5], thus affecting microbial functions and subsequently ecological C and nitrogen (N) cycling processes [6]. Therefore, in addition to directly participating in litter degradation, protist communities may indirectly regulate litter degradation processes by influencing litter microbial communities.

Litter microorganisms secrete a large amount of cellulose ligninolytic enzymes and proteolytic enzymes, which together are generally regarded as the main driving force of litter degradation [7]. Cellulolytic enzymes are also referred to as cellulase, and cellulose is eventually hydrolyzed to glucose with the presence of cellulase [8]. Lignin is the most difficult component to degrade in litter, and its decomposition mainly depends on ligninolytic enzyme activity. At present, extensively studied ligninolytic enzymes include peroxidase, laccase, polyphenol oxidase, catalase, etc. [9] In the study of litter decomposition, protease and urease are the N-cycling enzymes which have received the most attention. At present, it has been identified that the vesicles and other such structural components are associated with the mechanism of osmotic nutrition in a variety of soil protists (i.e., amoeba, vesicle, etc.) [10] Some scholars have speculated that these protists may play a key role in the process of organic decomposition, just like bacteria and fungi [7]. Therefore, exploring the relationship between protozoan communities and activities associated with enzyme degradation in litter would be highly significant in our understanding of the specific role that they play.

The Zhongtiaoshan Northern Copper Mine is in Yuanqu County, Yuncheng City, Shanxi Province, which is one of the seven key copper producing regions of China. In this mine, copper tailings have continuously accumulated over time, which have seriously degraded local soil conditions. A previous study reported on a vegetation community grouping that had naturally recolonized the “Eighteenth” River tailings dam of the Northern Copper Mine, which included the grass species *Bothriochloa ischaemum* and *Imperata cylindrica* [11]. These two grass species have become dominant in multiple tailing sub-dams in the region, and both have produced significant amounts of litter following their introduction. Litter decomposition can therefore be used as a means of ecological restoration in this mining region, considering that this process is highly significant to soil nutrient cycling [12]. However, most studies on the microbial mechanisms of litter decomposition have mainly focused on bacterial and fungal communities [13,14]. That is to say, relatively few studies have explored how protists may influence such mechanisms. Accordingly, our study used litter from these two dominant grass species (*B. ischaemum* and *I. cylindrica*) that have recolonized this copper tailings dam as the experimental material. We used high-throughput sequencing and correlation analysis to determine protozoan community composition and diversity in litter and to compare differing protozoan community characteristics between these two grass species, as well as key influencing factors. We will test whether areas dominated by different grass species in mined areas harbor different protistan communities. Is there any key protozoan species play in their communities during litter de-composition processes of these two grass species? This study will provide a theoretical basis for the remediation of degraded ecosystems through microbial decomposition mechanisms in litter.

## 2. Materials and Methods

### 2.1. Site Description and Litter Sampling

The “Eighteenth” River copper tailing dam of the Northern Copper Mine was initially constructed in 1969 and was first put into operation in 1972. The tailings dam has expanded yearly as mineral sand deposits were covered by extraneous soil, and every 3–5 years a new sub-dam is produced based on the original sub-dam. At the time of this article, a total of 16 sub-dams have been constructed. This region has four distinct seasons under the influence of a continental monsoon climate, with an average annual temperature of 14 °C and an average annual precipitation rate of 780 mm (mainly during summer). Following years of restoration, flora has naturally recolonized this copper tailings dam. Among these flora, *B. ischaemum* and *I. cylindrica* have become the dominant species of the herbaceous layer.

### 2.2. Litter Sample Collection

In April 2019, litter samples were collected in three plots at S536 sub-dam (which correspond to 22 years of restoration). We collected one *I. cylindrical* litter sample and one litter *B. ischaemum* sample in each 1 × 1 m plot at S536 sub-dams. The distance between each plot was greater than 50 m. Six litter samples were collected from three experimental plots. Sterile gloves were worn during the entire sampling process to prevent sample contamination. The collected samples were packed in sterile plastic bags, placed in ice boxes, and immediately transported to the laboratory. Each litter sample was divided into three parts: one part was stored at 4 °C to determine enzyme activity degradation; one part was oven dried (60 °C) to a constant weight to determine litter nutrient content; one part was stored at −20 °C for use with high-throughput sequencing.

### 2.3. Chemical Properties and Enzyme Activities in Litter

An elemental analyzer (vario EL/MACRO cube, Elementar Analysensysteme GmbH, Hanau, Germany) was used to measure total carbon (TC) and total nitrogen (TN) in litter samples. Potassium permanganate titration was used to measure catalase activity, where catalase activity was expressed per mg of hydrogen peroxide (H_2_O_2_) and was decomposed per g of litter over a 20 min period. 3,5-Dinitrosalicylic acid colorimetry was used to measure sucrase and cellulase activity, where sucrase activity was expressed as per mg of glucose produced by 1 g of litter after 24 h and where cellulase activity was expressed as per mg of glucose produced by 1 g of litter after 72 h. Sodium phenolate-sodium hypochlorite colorimetry was used to measure urease activity, where urease activity was expressed as per mg of ammonium nitrogen produced by 1 g of litter after 24 h. Iodometric titration was used to measure polyphenol oxidase activity, where polyphenol oxidase activity was equivalent to 0.01 mol/L I2 of the litter filtrate.

### 2.4. DNA Extraction Using High-Throughput Sequencing

The E.Z.N.A. soil DNA Kit (Omega Bio-Tek Inc., Norcross, GA, USA) was used to extract DNA in litter, and a 0.8% agarose gel (electrophoresis) was used to examine DNA quality and size. The universal eukaryotic primers TAReuk454FWD1F/TAReukREV3R9 (5′-CCAGCASCYGCGGTAATTCC-3′/5′-ACTTTCGTTCTTGATYRA-3′) were used to amplify the V4 hypervariable region of the 18S rDNA gene [15]. A 30 μL reaction system was used for polymerase chain reaction (PCR): the 15 μLPhusion^®^ High-Fidelity PCR MasterMix (New England BioLabs, Ipswich, MA, USA), 0.2 μ mol/L primer, and 10 ng DNA template. PCR preconditions were as follows: 98 °C for 1 min; 98 °C for 10 s, 50 °C for 30 s, 72 °C for 60 s, 30 cycles, 72 °C for 5 min. The NanoDrop ND-1000 UV-Vis Spectrophotometer (NanoDrop Technologies, Wilmington, DE, USA) was used to purify PCR products. Finally, paired-end sequenced on an Illumina MiSeq PE300 platform (Illumina, San Diego, CA, USA) according to the standard protocols by Majorbio Bio-Pharm Technology Co. Ltd. (Shanghai, China).

### 2.5. Sequence Processing and Taxonomic Classification

Trimmomatic software was used to integrate the original sequencing data in FASTQ format. UPARSE software (version 7.1, http://drive5.com/uparse/, accessed on 23 March 2020) was used to cluster high-quality sequences (operational taxonomic units, OTU) under 97% similarity [16]. To obtain the species classification information that corresponds to each OTU, an RDP classifier Bayesian algorithm was used to compare representative OTU sequences, and the PR2 database (version 4.5) was used with a reliability threshold of 70%. Plant (Streptophyta), animal (Metazoa), and fungal sequences were also removed [17] before being flattened according to the minimum number of sample sequences required to generate retained and conserved protozoan OTU tables.

### 2.6. Statistical Analysis

An independent sample t-test (conducted in SPSS 24.0) was used to analyze physical and chemical characteristic differences between the two different plant litters. R3.5.3 was used for Venn diagram and community composition analyses. The community composition of protozoa in the different litter samples was analyzed at an order and genus level, and significant differences between the top 10 dominant protozoa groups were tested. The Shannon–Wiener index, the Simpson index, the Chao1 index, the ACE index, and the Coverage index were used to reflect protozoan diversity, while the diversity index of protozoa in the different plant litter was also analyzed. Spearman’s rank correlation coefficient was used to explore correlations between the diversity index, the richness index, and the physical and chemical properties of litter. In addition, Spearman’s rank correlation analysis was conducted between the top 25 dominant groups with a relative abundance as well as the physical and chemical properties of litter at an order and genus level. R3.5.3 was used for the abovementioned analyses. Finally, linear discriminant analysis (LDA) was conducted according to different grouping conditions by means of Linear discriminant analysis Effect size (LEfSe) analysis to determine which communities or species exhibited significant differences in the classification of samples. The LDA threshold used in this study was 2.

## 3. Results

### 3.1. Physical and Chemical Properties of Natural Grass Litter

Results showed some differences in the physical and chemical properties of plant litter. For nutrient characteristics, the TC content of *B. ischaemum* litter was significantly higher than that of the *I. cylindrica* litter; however, we found no significant differences in TN content and the C/N ratio between the two grass species. For enzyme activities, only urease activity in *B. ischaemum* litter was significantly lower than that in *I. cylindrica* litter. Other enzyme activities were higher than that of *I. cylindrica* litter, and cellulase activity was significantly higher than that of *I. cylindrica* litter (Table 1).

### 3.2. Community Diversity of Litter Protozoa

The average length sequencing was 378.70 bp. The Good’s coverage of protozoan communities was greater than 99.5% in both *I. cylindrica* and *B. ischaemum* litter (Table 2). At a 97% similarity level, 101 OTUs were obtained. Among these, 15 and 39 OTUs were unique to *B. ischaemum* litter and *I. cylindrica* litter, respectively, and 47 OTUs were common to both, indicating that the protozoan communities in *B. ischaemum* litter and *I. cylindrica* litter were similar. Results also showed that the Shannon–Weiner index of *B. ischaemum* litter was higher than that of *I. cylindrica* litter, while the Simpson index, the ACE index, and the Chao1 index were lower; however, we detected no significant differences in diversity between the different protozoan communities in the litter (*p* > 0.05) (Table 2).

### 3.3. Community Composition of Litter Protozoa

**Figure 1:**
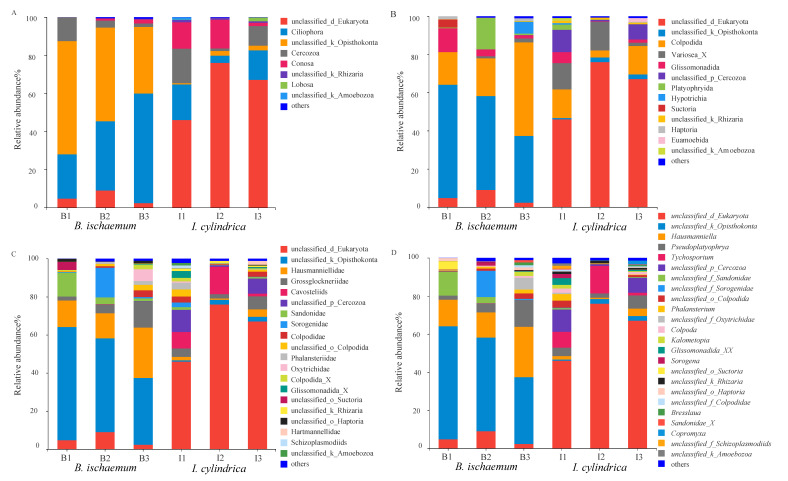
Composition of protist communities in litters at different taxonomic levels. (**A**): Phylum. (**B**): Order. (**C**): Family. (**D**): Genus level. B1, B2 and B3 represent litter samples of *B. ischaemum.* I1, I2 and I3 represent litter samples of *I. cylindrica*.

The protozoan communities detected in *B. ischaemum* and *I. cylindrica* litter belonged to 8 kingdoms, 13 phyla, 21 classes, 30 orders, 42 families, 50 genera and 53 species. Results from species composition analysis showed significant differences in the protozoan composition between *I. cylindrica* and *B. ischaemum* litter (Figure 1). At a phylum level, the relative abundance of unclassified_k_Opisthokonta was the highest (47.87%), followed by Ciliophora (39.06%) and Cercozoa (6.01%). Most the litter protozoa were unclassified_d_Eukaryota (62.99%), followed by Ciliophora (12.66%) and Conosa (10.22%). At an order level, the position of Colpodida showed dominance in both grass species, with a relative abundance of 28.64% and 11.21%, respectively. Glissomonadida was the main protozoan group (5.91%) in *B. ischaemum* litter, while the relative abundance of variosea_X in *I. cylindrica* was higher (10.19%) (Figure 1A). Results from our intergroup difference test showed significant differences between unclassified_Eukaryotes and unclassified_Opisthokonta between the two grass species (*p* < 0.01) (Figure 1B). In addition, *Hausmanniella* and *Tychosporium* were the dominant species in *B. ischaemum* and *I. cylindrica* litter, respectively, while the relative abundance of *Hausmanniella* differed significantly between the two grass species (Figure 1D).

### 3.4. Difference Analysis of Litter Protozoa Community

**Figure 2:**
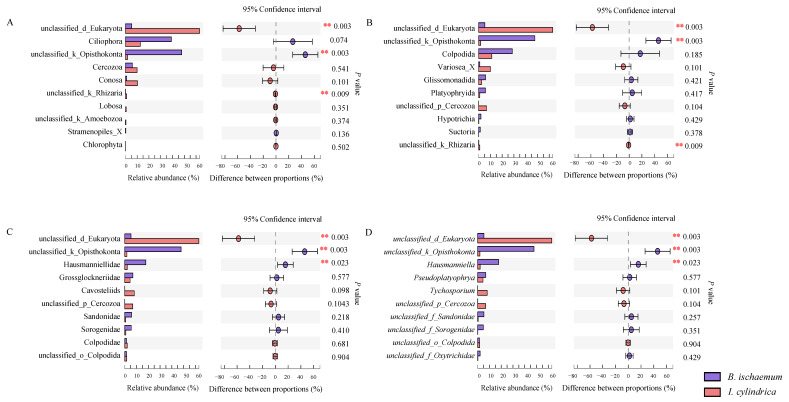
Difference analysis of dominant protist groups among different litters. The top 10 dominant group in relative abundance at the level of phylum (**A**), order (**B**), family (**C**), genus (**D**).

The results showed that there were significant differences in the relative abundance of unclassified_Eukaryotes and unclassified_Opisthokonta among different litter types (*p* < 0.01) (Figure 2). At the family and genus level, the relative abundance of Hausmanniellidae in the litter of *B. ischaemum* was significantly higher than that of *I. cylindrica* (*p* = 0.023) (Figure 2C,D).

**Figure 3:**
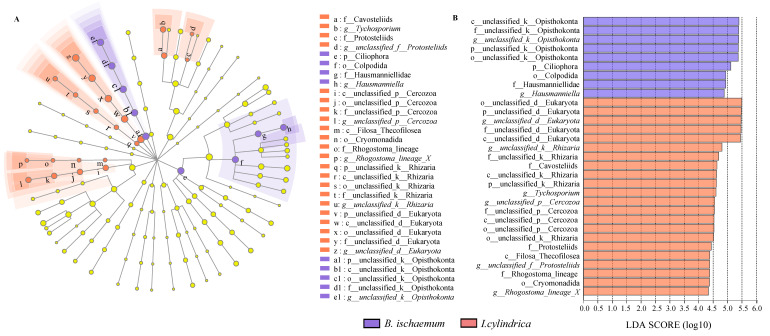
LEfSe analysis of protist communities among the two litter types (**A**): LEfSe analysis of protist communities with LDA scores greater than 2.0. Different color nodes indicate a significant enrichment in corresponding groups, while yellow nodes indicate no significant differences between groups. (**B**): LDA bars represent protist communities among the two litter types with LDA scores greater than 2.0.

LEfSe results showed 31 different protozoan groups in *I. cylindrica* and *B. ischaemum* litter at a genus level, which included 9 in *B. ischaemum* litter and 22 in *I. cylindrica* litter. In *B. ischaemum* litter, *Hausmanniella* (LDA: 4.88) and unclassified_Opisthokonta (LDA: 5.38) were the protozoa detected at a genus level. The main protozoan types found in litter were *Tychosporium* (LDA: 4.34), *Rhogostoma_lineage_X* (LDA: 4.34), and *unclassified_f_Protostelids* (LDA: 4.38) (Figure 3).

### 3.5. Litter Characteristic Effects on Protozoan Communities

**Figure 4:**
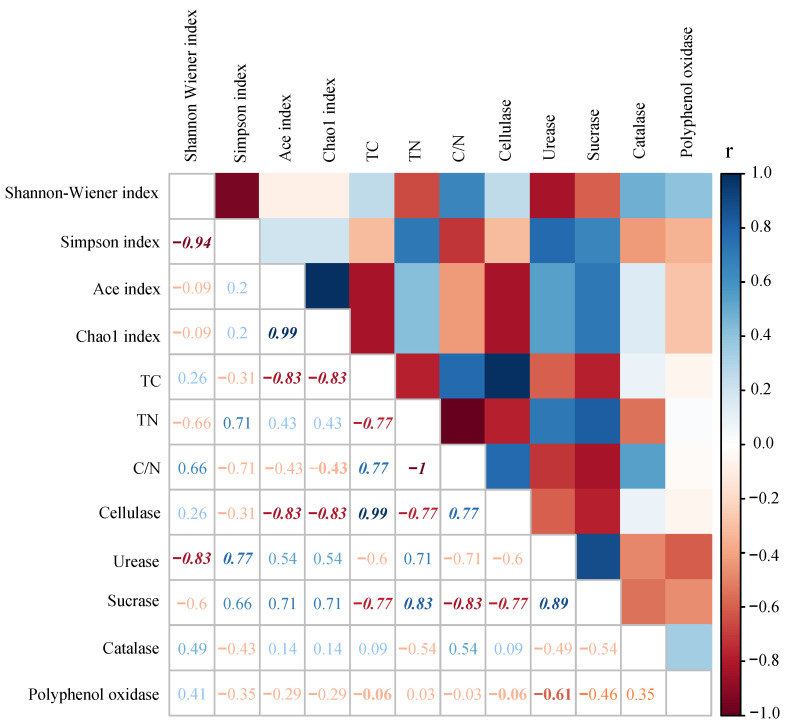
Spearman’s rank correlation analysis between diversity indices of protist communities and litter properties. Italics represent significant differences.

Results showed that the Shannon–Wiener index negatively correlated to urease activity, while the Simpson index positively correlated to urease activity. Protozoan community richness indices (i.e., the ACE index and the Chao1 index) negatively correlated to TC and cellulase activity in litter (Figure 4).

**Figure 5:**
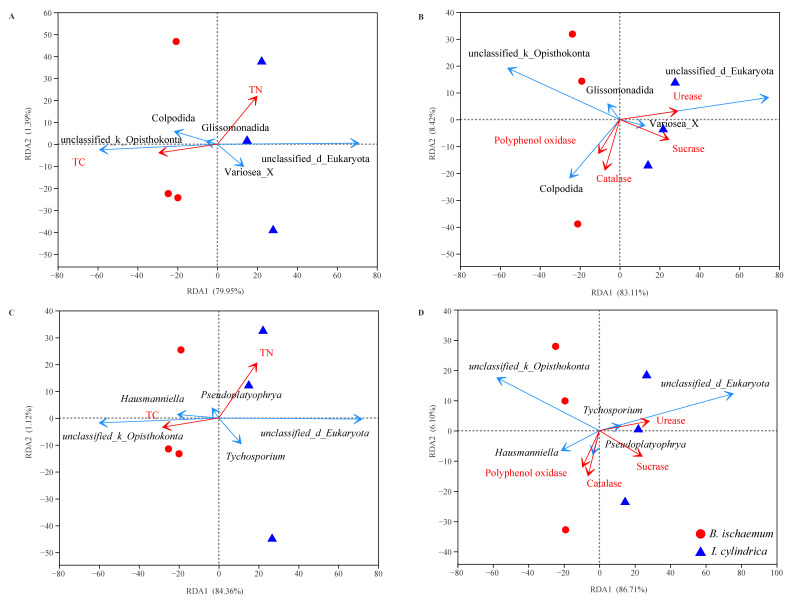
Redundancy analysis to determine correlations between protist communities and litter properties. Correlations between protist communities and contents of nutrient characteristics (**A**) and enzymatic activity (**B**) at an order level. Correlations between protist communities and contents of nutrient characteristics (**C**) and enzymatic activity (**D**) at the genus level. The red arrow indicates litter properties and the blue arrow indicates the dominant groups with the top 5 relative abundance.

The environmental factors were screened by variance expansion factor (VIF) analysis. The results showed that the main ecological factors affecting the protozoa community were different between the different litters. The litter protist communities of *B. ischaemum* was mainly affected by the TC of the litter, while the TN, urease activity and sucrase activity were the important ecological factors affecting the protist communities of *I. cylindrica* litter (Figure 5).

**Figure 6:**
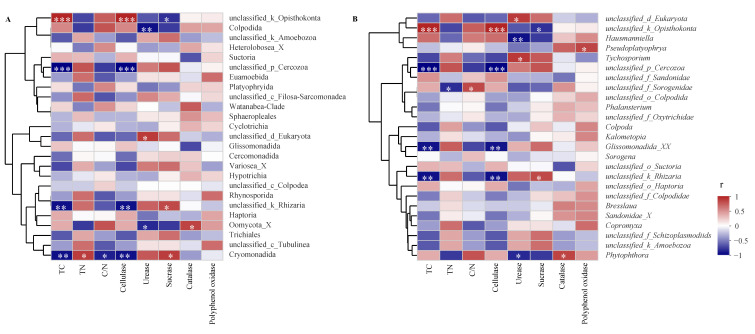
Spearman’s rank correlation analysis between the dominant protists and litter properties at an order (**A**) and a genus (**B**) level (* *p* < 005; ** *p* < 0.01; *** *p* < 0.001).

At both an order and genus level, we selected the top 25 dominant protozoa communities (with relative abundance) and litter properties for correlation analysis. Results showed that most protozoa significantly and was positively correlated to TN content and urease and invertase activities, while they negatively correlated to TC content and cellulase and catalase activities (Figure 6A). Specifically, Cryomonadida positively correlated to TN content and invertase activity (*p* < 0.05) and was negatively correlated to TC and cellulase activity (*p* < 0.01). On the other hand, Colpodida and Oomycota_X negatively correlated to litter urease activity (*p* < 0.05). At a genus level, most protozoa were positively correlated to TN and polyphenol oxidase activity but were negatively correlated to TC content and cellulase and urease activities (Figure 6B). The following describes the specific performance observed: *Tychosporium* was significantly and positively correlated to urease activity (*p* < 0.05), and *Glissonadida*_XX was negatively (significantly) correlated to TC content and cellulase activity (*p* < 0.01). *Phytophthora* was negatively correlated to urease activity and was positively correlated to catalase activity (*p* < 0.05). In addition, there was a significant positive correlation between *Pseudoplatyophya* and polyphenol oxidase activity (*p* < 0.05).

## 4. Discussion

Many protists reside in both plant leaves, as well as in a variety of species [18], which has an effect on bacterial community characteristics and which alter microbial community functionality [7]. Results from this study showed that ciliates and Cercozoa were the two dominant protozoan communities in both litter types. Ning et al. [19,20] analyzed ciliate diversity in the forest soil of Mount Taibai and in the swamp and wetland areas of the Gannan Plateau, China. They found that Colpodida was common to both environments as well as being the dominant community in heavy metal contaminated soil, which indicated that Colpodida has a certain tolerance to heavy metal pollution [21]. Furthermore, Bonanomia et al. [22] reported that ciliate and amoeba were the main protozoan communities found in a variety of plant litter. Ciliates are also an important component of microbial communities and inhabit almost all environments on earth. They play important roles in regulating bacterial communities and transforming nutrients [23]. Results from this study showed a difference in protozoan composition between *I. cylindrica* and *B. ischaemum* litter. For example, the dominant *Hausmanniella* species group significantly differed between the litter of these two grass species, and the relative abundance of *Tychosporium* and *Rhogostoma_lineage_X* in the *I. cylindrica* litter was significantly higher than in the *B. ischaemum* litter. Among these, Tychosporium, being morphologically similar to *Protostelium mycophaga*, is a typical mononuclear amoebae that mainly feeds on bacteria [24]. Besides preying on bacteria, *Rhogostoma* also plays a role in regulating eukaryotic communities [18]. Some studies have found that protozoa do not feed on all bacteria equally, while they have a preference for bacteria and fungi [25]. The feeding preference of protozoa has a direct effect on community dynamics and colony aggregations of bacteria and fungi, and further affects expressions related to associative enzyme degradation processes. Therefore, it is necessary for future studies to investigate protist, bacteria, and fungi community behavior in combination. This will provide a basis to further explore the roles that protists play in litter decomposition.

In addition to impacting bacterial and fungal communities, certain protists also participate in the degradation of organic matter and play an important role in C and N cycling and nutrient transformation processes [26]. For example, oomycetes can promote the degradation of organic matter through lysotrophic processes [27]. Results from this study showed that amoebic Euamoebida and foraminiferous Cryomonadida both significantly and positively correlated to urease and sucrase activities in litter, and they may also be involved in litter decomposition processes. Vesicular Colpodida and Oomycota_X stramenopiles both strongly correlate to catalase and polyphenol oxidase, and these extracellular enzymes belong to ligninolytic enzymes [28], indicating that protist groups are significant in the degradation of refractory C sources, such as lignin.

Although protozoan diversity in *B. ischaemum* litter was higher than in *I. cylindrica* litter, its overall richness was lower, which may be due to the lower C/N ratio of *I. cylindrica* litter. Protozoa mainly fed on soil bacteria [26], and litter quality leads to changes in microbial community composition. Fungal propagation favors a high C/N ratio, while bacterial propagation favors a low C/N ratio [29,30]. Findings from this study indicated that the C/N ratio of *I. cylindrica* litter was comparatively lower, which was more conducive to the growth of bacterial communities, thus providing adequate food for the growth of phagocytic-type protists. Studies have reported that protist functionality is limited by water conditions [31], and those that reside in the interleaf of plants are typically more active at night, when dew has accumulated on leaves [32]. This study found no significant difference in protozoan diversity in *B. ischaemum* and *I. cylindrica* litter, which may be because both litter (plant) types were derived from the same sub-dam. Similarities in external environmental conditions may be an important reason for the slight differences found in protozoan community diversity between the two litter types, but the composition of different protozoan communities in litter differed significantly. This could be due to differences in the litter properties and root exudates of the two grass species or microenvironmental changes. This could also be due to the different influence of the bacterial and fungal communities. All these factors can affect the composition of the protozoan community [33].

Community characteristics of protists are typically affected by a variety of ecological factors and soil nutrients (i.e., C and N) are key factors that regulate the diversity, density, and community composition of soil protozoa [34]. Krashevska et al. [35,36] found that additive C and phosphorus (P) will reduce the diversity and density of shell-bearing amoebas, while additive N had the opposite effect. At the same time, the C/N ratio of protists is higher than bacteria, and they are only behind phagocytic bacteria in excreting excess N [37], which subsequently increases soil fertility [38]. Similarly, this study also found that protist richness was significantly and negatively correlated to TC content in litter, and, at an order level, most dominant groups were negatively correlated to litter C content, while they were positively correlated with TN content. Compared to soil ecosystems, however, our understanding of protist behavior in litter is limited, particularly in degraded or damaged ecosystems, wherein the response of protist communities to environmental change may be more complex [39]. Thus, it is necessary to further explore the relationships between litter properties and protozoan communities in combination with the chemical composition of litter, as well as protozoan community succession in degraded ecosystems.

## Figures and Tables

**Table 1:** Plant litter properties (values denote means with standard deviations).

	TC (%)	TN (%)	C/N	Cellulase (mg·(g·72 h)^−1^)	Urease (mg·(g·24 h)^−1^)	Sucrase (mg·(g·24 h)^−1^)	Catalase (mg·(g·20 min)^−1^)	Polyphenol Oxidase (mL·g^−1^)
*B. ischaemum*	44.442 ± 0.057a	0.468 ± 0.007	95.000 ± 1.512	1.200 ± 0.077a	0.969 ± 0.478b	1.395 ± 0.047	3.607 ± 0.866	5.867 ± 0.321
*I. cylindrica*	43.157 ± 0.113b	0.583 ± 0.097	75.377 ± 12.721	0.772 ± 0.054b	3.760 ± 0.308a	3.072 ± 0.716	3.237 ± 0.231	5.500 ± 1.000

Note: Different lowercase letters indicate significant differences (*p* < 0.05), while values without letters indicate no significant differences.

**Table 2:** Diversity index results of protozoan communities in plant litter.

	Coverage	Shannon–Wiener Index	Simpson Index	Chao1 Index	ACE Index
*B. ischaemum*	0.997 ± 0.001	2.318 ± 0.454	0.178 ± 0.097	47.083 ± 15.261	48.467 ± 15.984
*I. cylindrica*	0.995 ± 0.001	1.733 ± 0.602	0.414 ± 0.178	66.917 ± 14.036	66.763 ± 10.907

## Data Availability

Not applicable.
